# RAB-10 Regulates Dendritic Branching by Balancing Dendritic Transport

**DOI:** 10.1371/journal.pgen.1005695

**Published:** 2015-12-03

**Authors:** Caitlin A. Taylor, Jing Yan, Audrey S. Howell, Xintong Dong, Kang Shen

**Affiliations:** Howard Hughes Medical Institute, Department of Biology, Stanford University, Stanford, California, United States of America; University of California San Diego, UNITED STATES

## Abstract

The construction of a large dendritic arbor requires robust growth and the precise delivery of membrane and protein cargoes to specific subcellular regions of the developing dendrite. How the microtubule-based vesicular trafficking and sorting systems are regulated to distribute these dendritic development factors throughout the dendrite is not well understood. Here we identify the small GTPase RAB-10 and the exocyst complex as critical regulators of dendrite morphogenesis and patterning in the *C*. *elegans* sensory neuron PVD. In *rab-10* mutants, PVD dendritic branches are reduced in the posterior region of the cell but are excessive in the distal anterior region of the cell. We also demonstrate that the dendritic branch distribution within PVD depends on the balance between the molecular motors kinesin-1/UNC-116 and dynein, and we propose that RAB-10 regulates dendrite morphology by balancing the activity of these motors to appropriately distribute branching factors, including the transmembrane receptor DMA-1.

## Author Summary

Building a complex dendritic arbor requires tremendous cellular growth, and how membrane and protein components are transported to support a rapidly growing, polarized dendrite remains unclear. We have identified the small GTPase RAB-10 as a key regulator of this process, providing insight into both dendritic development and the control of trafficking by small GTPases.

## Introduction

There is great diversity in the structure and complexity of dendritic arbors across neuron types, and establishing the correct dendritic morphology is critical for the proper connectivity and function of neural circuits. A developing dendritic arbor must target a specific receptive field, adopt the appropriate neuron-specific architecture, and avoid overlapping in connectivity with itself and neighboring dendrites. A number of extrinsic cues and intrinsic mechanisms help orchestrate the formation of these complex neuronal morphologies, including transcriptional programs, extracellular guidance cues, and contact-dependent repulsive molecules that mediate self-avoidance [1–5].

Dendritic arbor development requires tremendous cellular growth and likely has specialized membrane trafficking demands. Little is known about how the transport of branching factors and membrane components is coordinated across a large, polarized neuron. Dendrites are more sensitive than axons in their reliance on the membrane supply from secretory pathways [2,6], and they have distinct transport needs. For example, a set of dendritic arbor reduction (*dar*) genes have been identified which are required for dendritic arbor outgrowth but not axon growth. These *dar* genes are important for ER-to-Golgi transport [6]. In addition, the Rab GTPases, a conserved family of small GTPase proteins that regulate membrane identity and vesicle trafficking [7–9], are likely important for the polarization and outgrowth of neurites, though their precise role in both axonal and dendritic development remains unclear [10].

One of these small GTPases, Rab10, has been shown to mediate membrane trafficking in several polarized cell types, including neurons [11–18]. The importance of Rab10 for endosomal sorting and endocytic recycling has been demonstrated in *Drosophila* epithelial cells [15] as well as in *C*. *elegans* neurons [16–17] and intestinal epithelial cells [11–14]. In hippocampal neurons, Rab10 is required for directional membrane trafficking in the growing axon [19–20], and it has been shown to directly bind the anterograde motor kinesin1 via the adaptor protein JIP1 [21]. In addition to sorting and trafficking cargo to the appropriate destinations, a growing neurite must appropriately dock and release membrane and protein cargoes. The docking of Rab10-postive vesicles is required for axon outgrowth in hippocampal neurons [22]. Rab10 has also been linked to the exocyst, a secretory complex responsible for polarized exocytosis [23–25].

The exocyst is an octameric complex that was initially identified in yeast for its role in membrane addition during bud outgrowth, and it has since been shown to have conserved functions in polarized growth across several cell types [25–28]. In *Drosophila*, the *sec5* component of the exocyst complex is required to establish polarity in the oocyte [26], and the *sec15* component is also required for the polarized delivery of photoreceptors [28]. Both Rab10 and the exocyst complex are required for branch outgrowth in the *Drosophila* trachea [25]. Taken together, these data suggest that both the exocyst complex and RAB-10 support outgrowth in polarized cells; however, the roles of RAB-10 and the exocyst have never been examined in a growing dendrite.

In *Caenorhabditis elegans*, the PVD sensory neuron forms elaborate dendritic arbors that are organized into orthogonal tiers of characteristic menorah-shaped units [29–30]. The PVD neuron has proved to be useful in the study of the molecular mechanisms of dendrite morphogenesis. Importantly, the dendritic branches of PVD maintain self-avoidance [31], a key feature across neural networks. Extracellular cues are essential for PVD dendritic morphogenesis: the branching receptor DMA-1, expressed in PVD, instructs spatially-restricted growth of dendritic branches via interaction with the SAX-7/MNR-1 adhesion complex [32–34]. Previous work has also identified various intracellular processes important for PVD dendritic morphology, including transcription factors that instruct cell fate [29,35], microtubule-associated proteins [36], and membrane fusion proteins that prevent over-branching [37]. However, the role of polarized membrane trafficking in the growing PVD dendrite remains uncharacterized.

In this work, we identified a novel role for the small GTPase RAB-10 in patterning the dendritic arbor of PVD. We demonstrate that both RAB-10 and the exocyst complex are required for proper outgrowth and patterning of the PVD dendritic arbor. RAB-10 and EXOC-8 are also required for the appropriate localization of branching factors such as DMA-1. Additionally, we found that the molecular motors kinesin and dynein are important for anterior-posterior patterning of the PVD dendritic arbor. The PVD dendritic outgrowth phenotype was also observed in a recently published report [38], and our work further identifies a role for RAB-10 in dendritic patterning beyond its role in promoting outgrowth. We propose that RAB-10 regulates dendrite morphogenesis by balancing anterograde and retrograde transport via molecular motors.

## Results

### *wy787* disrupts dendritic arbor morphology

**Fig 1:**
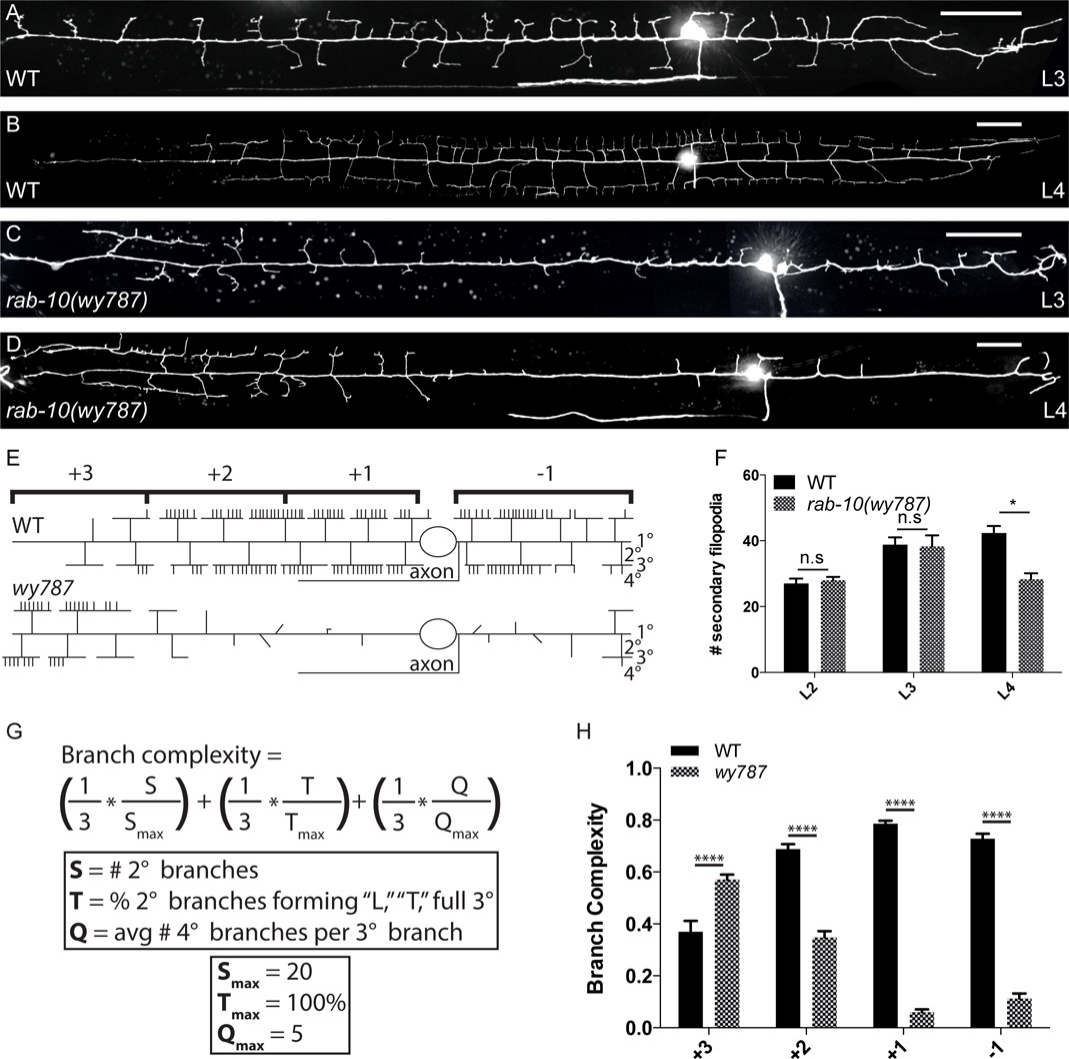
*rab-10(wy787)* causes defects in PVD dendritic morphology. (A, B) PVD was labeled with a cell-specific marker (*ser2prom3*::*myrGFP*). The dendritic arbor covers the entire body of a wild-type animal. 2° and 3° branches are established by the L3 larval stage, and by the L4 larval stage the dendritic arbor has fully elaborated with 4° branches. A second cell body appears in some micrographs because *ser2prom3*::*myrGFP* also labels the non-branching PDE neuron. (C, D) *wy787* mutants have severely disrupted dendritic morphology. 2° branches emerge in the L3 stage but most do not reach the 3° branch-point or extend 3° branches. Full menorah form only in the distal anterior region of the dendrite. (E) Schematic of the PVD dendritic arbor in wild-type and *wy787* animals. (F) Quantification of 2° branch filopodia in larval stages in wild-type and *wy787* animals. (G) Definition of branch complexity index used to quantify menorah completeness per region for each genotype. (H) Quantification of subcellular distribution of branch complexity in wild-type and *wy787* animals. Scale bars represent 20 μm. Error bars represent SEM. * p<0.05, **p<0.01, ***p<0.001, ****p<0.0001 by 2-way ANOVA with post-hoc Tukey’s multiple comparisons test. N>5 for all genotypes.

We visualized the morphology of the PVD neurons by expressing membrane-bound-GFP under the control of a cell-specific promoter (*ser2prom3*:*myrGFP*) (Fig 1A and 1B) [29]. In wild-type animals, one anterior and one posterior primary dendritic branch extend from the cell body during the L2 larval stage. Dense menorahs consisting of a series of orthogonal 2°, 3°, and 4° branches emerge from the anterior and posterior 1° dendrites as the animal progresses through subsequent larval stages (L2-L4) (Fig 1A, 1B and 1E). To understand the molecular mechanisms of dendritic branching, we performed a forward genetic screen to identify mutations that affect the dendritic morphology of PVD. From this screen, we isolated individuals with the fully penetrant and recessive mutant allele *wy787*, which show a severe reduction in the number of dendritic branches (Fig 1C and 1D). *wy787* mutant animals also have several non-neuronal phenotypes, including intestinal vacuoles and reduced brood size. In *wy787* mutants, the number of secondary branches is reduced from 42 ± 6 in wild-type to 28 ± 9 in *wy787*, and the number of quaternary branches is reduced from 114 ± 15 to 17 ± 10 (S1 Table). In addition to the reduction in branch number, we noticed a shift in branch distribution. In *wy787* mutants, considerably more branches are found in the distal anterior region of the 1° dendrite, where wild-type animals typically grow few branches (Fig 1B and 1D). To quantify the distribution of menorah, we divided the entire PVD dendritic arbor into four regions: the region posterior to the cell body (designated as “-1”) and three equal-length regions in the anterior dendrite (designated as “+1, +2 and +3”). We defined a “branch complexity” index to characterize the completeness of menorahs in each region. This branch complexity metric weights three characteristics equally: (1) the number of secondary branches, (2) the percentage of secondary branches that form a tertiary branch, (3) and the average number of quaternary branches per tertiary branch (Fig 1G). In *wy787* mutants, branch complexity is severely reduced in the +2, +1, and -1 regions but increased in the +3 region. (Fig 1C, 1D and 1H). The severe decrease in branch complexity in the +1 and -1 regions is the result of a near-total loss of 3° and 4° branches in the +1 and -1 regions (S1 Table). The increase in branch complexity in the anterior +3 region led us to hypothesize that the branching defects cannot be entirely explained by an overall lack of branching activity, but are instead the result of abnormal distribution of branching activity along the anterior-posterior axis.

The lack of branches in the posterior regions (+1 and -1) could be due to a defect in 2° branch outgrowth or stabilization. We distinguished between these possibilities by examining dendrite morphology during earlier developmental time points. In the L2 and L3 larval stages, wild-type and *wy787* showed no significant difference in the number of secondary filopodia (Fig 1F), which suggests that *wy787* could be defective in branch-stabilization. However, in *wy787* the posterior 2° branches are often shorter than they are in wild-type and often do not grow in the correct orthogonal orientation. These defective branches cannot reach the tertiary branch-point (Fig 1C), a region enriched for the branch-promoting complex, MNR-1/SAX-7, that supports tertiary branch outgrowth [33]. By the L4 stage, *wy787* had significantly fewer 2° branches, and those 2° branches that reached the tertiary line in the posterior regions (+1 and -1) failed to form 3° branches (Fig 1D and 1F). This developmental failure is consistent with a defect in both stabilization and outgrowth of 2° branches as well as both initiation and maintenance defects in 3° and 4° branches. This defective branching in the posterior regions is reminiscent of the phenotype of the branching receptor mutant *dma-1* [32–33].

### RAB-10 is required cell-autonomously for dendritic arbor morphology

**Fig 2:**
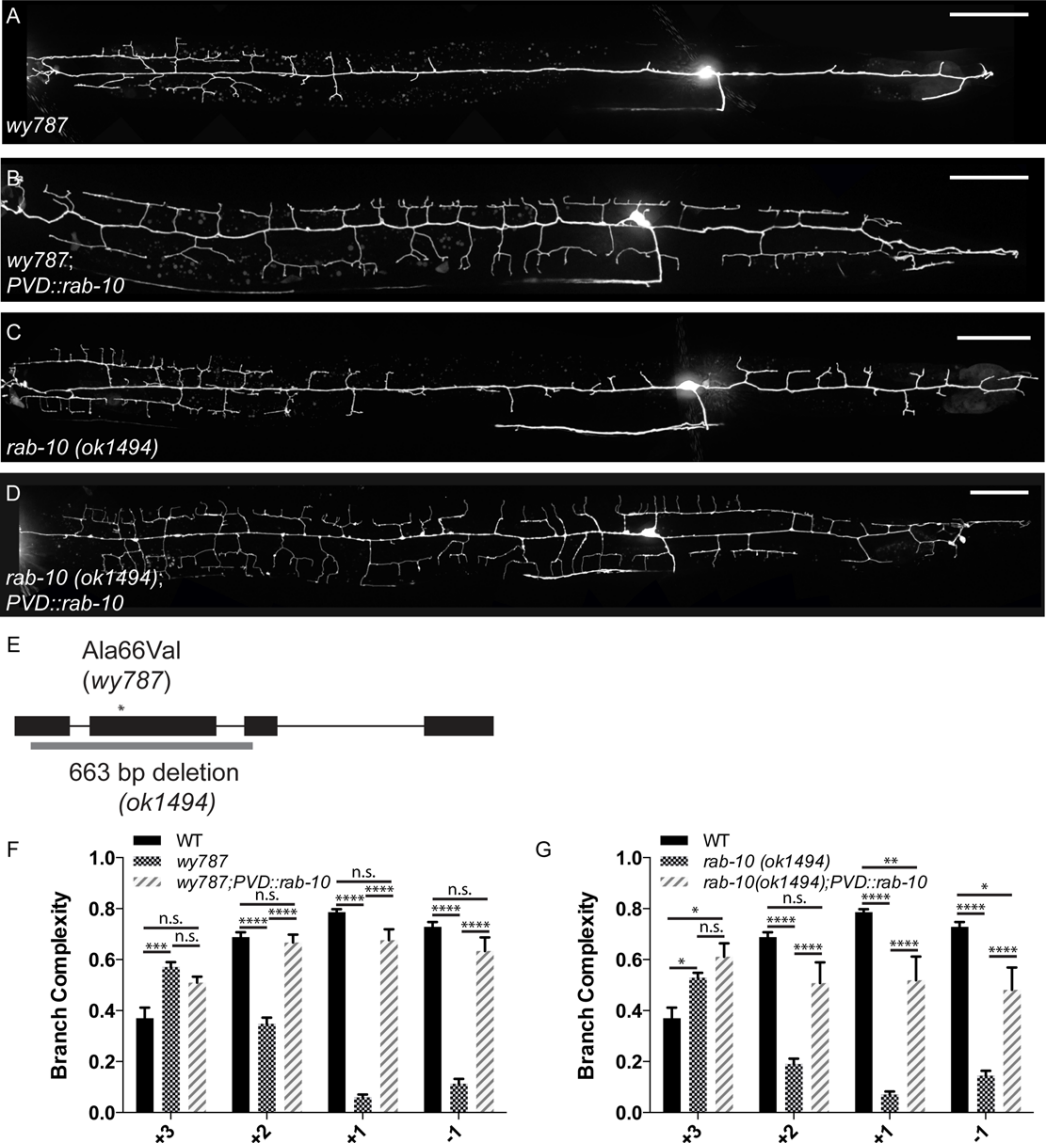
RAB-10 acts cell-autonomously to pattern the PVD dendritic arbor. (A-D) Representative images of PVD::GFP in various genotypes, as indicated on micrographs. (E) Schematic of *rab-10* genomic structure. The *ok1494* allele is a 663 base pair deletion. The *wy787* allele, indicated with an asterisk, is a missense Ala66Val mutation. (F,G) Quantification of subcellular distribution of branch complexity in wild-type, *wy787*, and *wy787;PVD*::*rab-10* animals (F) and wild-type, *ok1494*, and *ok1494;PVD*::*rab-10* animals (G). Scale bars represent 20 μm. Error bars represent SEM. * p<0.05, **p<0.01, ***p<0.001, ****p<0.0001 by 2-way ANOVA with post-hoc Tukey’s multiple comparisons test. N>5 for all genotypes.

We mapped and cloned the *wy787* allele and identified the causal lesion as a mutation in the small GTPase RAB-10. *wy787* contains a point mutation that results in a substitution of a conserved residue (A66V) in the GTP-binding domain of RAB-10 (S1 Fig) and can be rescued by *rab-10* genomic DNA (S1 Fig) and *rab-10* cDNA (Fig 2B). To confirm that RAB-10 is required for dendritic arbor growth, we also obtained the putative null allele *ok1494*, which has a 663 base pair deletion in the *rab-10* coding region and should cause a complete loss of function of this gene (Fig 2E). This deletion allele recapitulated the PVD dendritic phenotype of *wy787* with an even more severe reduction in posterior branch complexity and a more anterior shift in the +3 region compared with the *wy787* allele (Fig 2C).

To determine whether RAB-10 functions cell-autonomously within PVD to pattern the dendritic arbor, we expressed RAB-10 under the control of two PVD-specific promoters (*ser2prom3* and *pdes2short*) [30] in the *wy787* and *ok1494* mutant backgrounds. Expression of RAB-10 in PVD rescued the posterior reduction in branch complexity (Fig 2B, 2D, 2F and 2G), while muscle-specific expression of RAB-10 failed to rescue the dendritic phenotype of *rab-10* mutants (S1 Fig). Taken together, these data suggest that RAB-10 functions cell-autonomously to regulate the dendritic branch distribution of PVD neurons.

### The GTP-bound state of RAB-10 is required for dendritic arbor development

RAB-10 is a member of the Rab family of GTP-binding proteins. These conserved proteins help to control membrane identity and regulate intracellular vesicle trafficking [7–9]. A typical Rab GTPase adopts two conformations: an active GTP-bound form and an inactive GDP-bound form. Guanine nucleotide exchange factors (GEFs) help activate Rabs by catalyzing the exchange of GDP for GTP, and GTPase activating proteins (GAPs) accelerate the GTPase activity of Rabs [7]. The missense mutation in *wy787*, A66V, is two amino acids away from the GTP binding site of RAB-10 and likely interferes with the innate activity of RAB-10.

GTP-binding sites and GTPase sites are highly conserved across Rab GTPases in many species (S1 Fig), and it has been well established that these sites can be mutated to generate dominant-negative and constitutively active forms of Rab GTPases [11–14,17–18,20,23]. To assess whether the GTPase activity of RAB-10 is required for normal PVD development, we generated the constitutively active and inactive forms of RAB-10. The constitutively active mutant, Q68L, alters the GTPase site to prevent GTP-hydrolysis, thus locking RAB-10 in its active conformation. The inactive form, T23N, prevents GTP-binding and should result in an inactive RAB-10. Using a PVD-specific promoter, we expressed either the active or inactive form of RAB-10 in both wild-type and the *rab-10* (*ok1494*) mutant background. In the wild-type background, the putative GDP-locked (T23N) of RAB-10 likely acts as a dominant negative. We observed a moderate reduction in posterior branch complexity in 30% of animals, and 1 in 50 animals have a branching phenotype indistinguishable from that of *rab-10* (S1 Fig). Interestingly, overexpression of *rab-10(GDP)* also causes an anterior shift in branch complexity. This provides further support for the cell-autonomous action of RAB-10.

**Fig 3:**
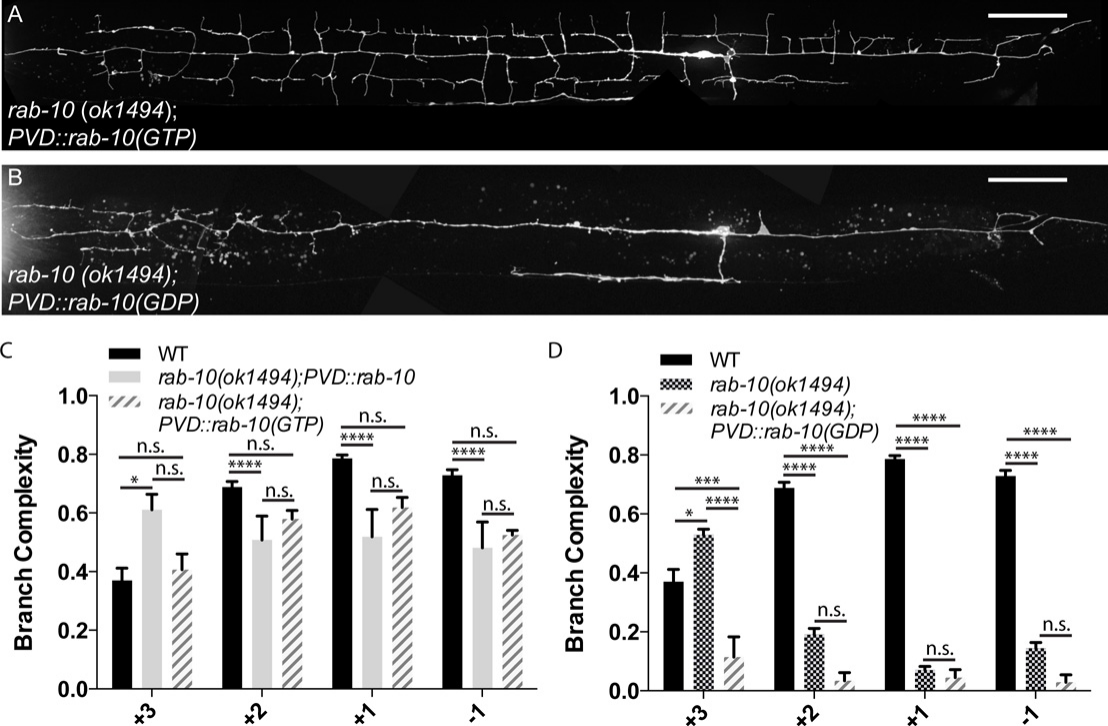
Active RAB-10 promotes dendritic branching, while inactive RAB-10 suppresses branching. (A,C) Representative image (A) and quantification (C) of subcellular distribution of branch complexity in *rab-10(ok1494)* expressing a constitutively active GTP-bound RAB-10 in PVD. (B,D) Representative image (B) and quantification (D) of subcellular distribution of branch complexity in *rab-10(ok1494)* expressing an inactive GDP-bound RAB-10 in PVD. Scale bars represent 20 μm. Error bars represent SEM. * p<0.05, **p<0.01, ***p<0.001, ****p<0.0001 by 2-way ANOVA with post-hoc Tukey’s multiple comparisons test. N>5 for all genotypes.

The putative GTP-locked form (Q68L) of RAB-10 rescued the dendritic morphology defect of *rab-10* (Fig 3A and 3C), though the rescue was not as robust as that of the wild-type form of RAB-10. The dominant-negative RAB-10(GDP) not only failed to rescue the dendritic defect but also further reduced branch complexity in the anterior region (Fig 3B and 3D). This is likely a dominant negative effect caused by sequestration of GEFs that interact with RAB-10 and one or more additional small G proteins. Together, these results support the notion that RAB-10 functions cell-autonomously as a GTPase to regulate PVD dendrite development.

**Fig 4:**
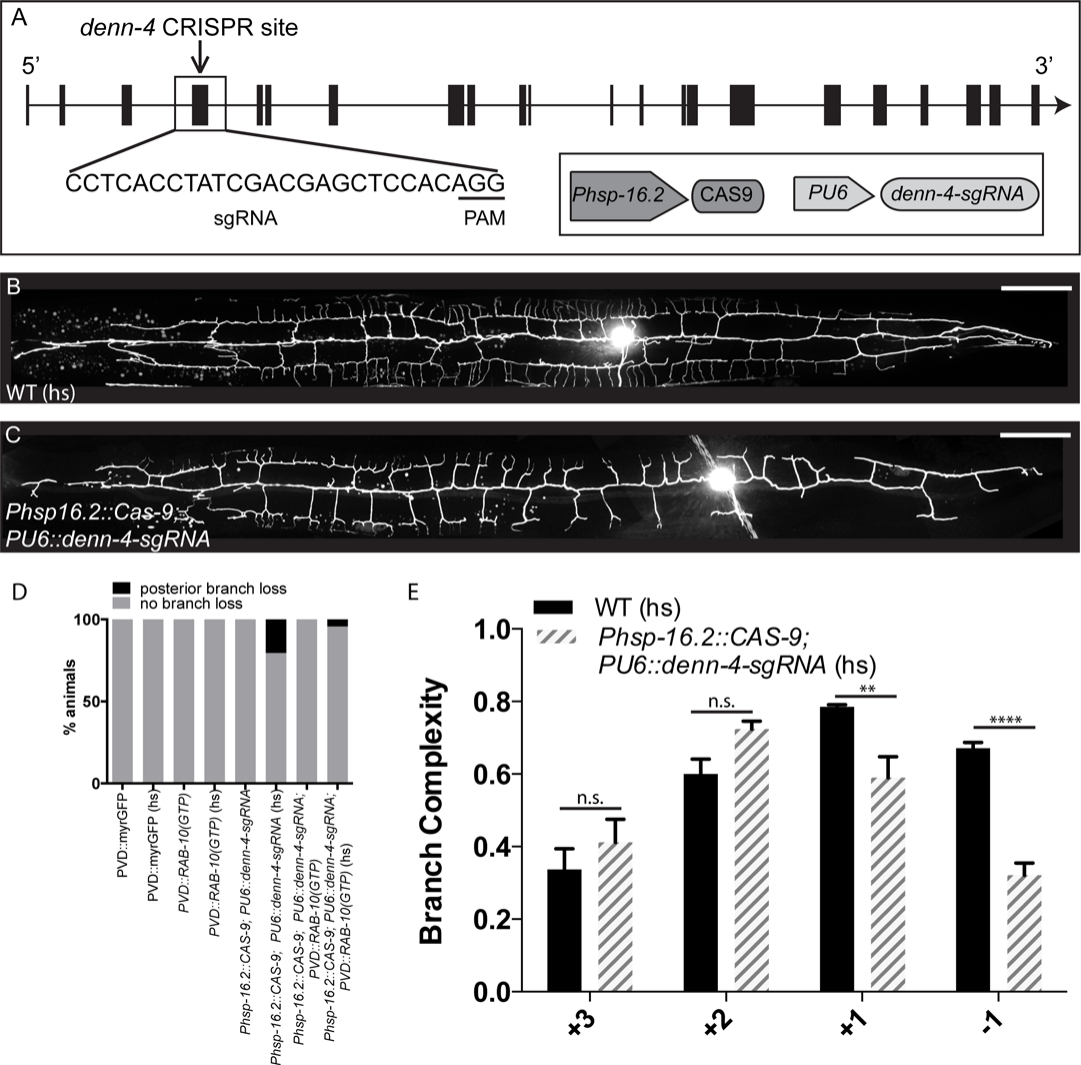
DENN-4, the putative RAB-10 GEF, is required for dendritic morphogenesis in PVD. (A) Schematic of somatic CRISPR targeting construct used to produce conditional *denn-4* mutations. (B) Representative image of PVD::GFP in a heat-shocked WT control animal. (C) Representative image of PVD::GFP in the conditional *denn-4* CRISPR allele. (D) Efficiency of conditional CRISPR *denn-4* knockout in generating PVD-phenotype in all conditions and genotypes tested. (E) Quantification of subcellular distribution of branch complexity in this allele. Scale bars represent 20 μm. Error bars represent SEM. * p<0.05, **p<0.01, ***p<0.001, ****p<0.0001 by 2-way ANOVA with Tukey’s multiple comparisons test. N≥3 for all genotypes.

The DennD4 protein has been shown to act as the GEF for the mammalian RAB10 protein [39]. We asked whether the worm DennD4 homolog, *denn-4*, is also required for PVD morphogenesis. Since the existing *denn-4* alleles result in early lethality, preventing us from studying PVD morphology, we used somatic CRISPR-Cas9 to generate a conditional *denn-*4 knockout (Fig 4A). We used a heat shock promoter (*Phsp16*.*2)* to drive the expression of CAS9 and an sgRNA designed to target *denn-4*, a method that has been proven effective in generating somatic mosaics [40]. *Phsp16*.*2* is active in many tissues, including neurons [41]. Indeed, knocking down *denn-4* with this strategy caused a reduction of branch complexity in the -1 and +1 regions in 21% of animals (Fig 4C–4E). While this phenotype is milder than the branch reduction seen in *rab-10*, it is qualitatively similar, suggesting that *denn-4* acts as a GEF for RAB-10 and to promote dendrite morphogenesis. In order to establish the relationship between *denn-4* and *rab-10*, we knocked down *denn-4* using somatic CRISPR-Cas9 while simultaneously overexpressing RAB-10(GTP) in PVD. Overexpression of RAB-10(GTP) reduced the penetrance of *denn-4* dendritic phenotypes to 6% (Fig 4D). Since *Phsp16*.*2* likely knocks down *denn-4* in many tissues, we cannot determine whether *denn-4* functions cell-autonomously within PVD. However, suppression of the *denn-*4 phenotype by PVD-specific expression of RAB-10(GTP) supports the notion that *denn-4* plays a role in dendritic development within PVD, and that *denn-4* acts as a GEF of *rab-10*, as suggested by studies in non-neuronal cells [39]. Together, these data argue strongly that the GTPase activity of RAB-10 is required to regulate dendrite morphogenesis in PVD.

### Mutations in the exocyst complex genes *exoc-8* and *sec-5* cause dendritic morphology defects similar to *rab-10*

**Fig 5:**
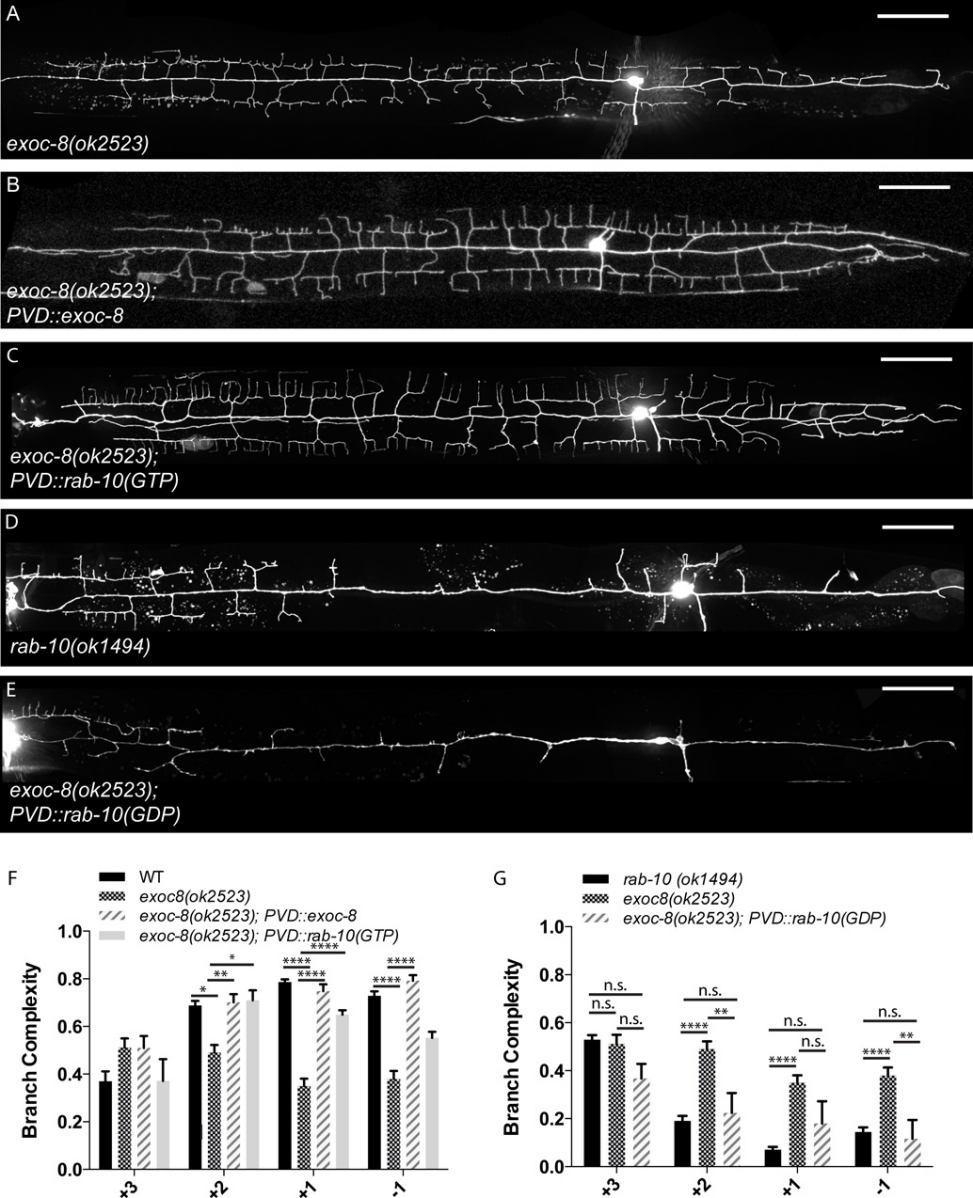
Mutations in the exocyst complex disrupt dendritic morphology. (A-E) Representative images of PVD::GFP in various genotypes as indicated on micrographs. (F) Quantification of subcellular distribution of branch complexity in wild-type, *exoc-8(ok2523)*, *exoc-8(ok253);PVD*::*exoc-8*, and *exoc-8(ok2523);PVD*::*RAB-10(GTP)* animals. Only significant relationships are noted; all unlabeled comparisons are not significant. (G) Quantification of subcellular distribution of branch complexity in wild-type, *exoc-8(ok2523)*, and *exoc-8(ok253);PVD*::*RAB-10(GDP)* animals. Scale bars represent 20 μm. Error bars represent SEM. * p<0.05, **p<0.01, ***p<0.001, ****p<0.0001 by 2-way ANOVA with Tukey’s multiple comparisons test. N≥5 for all genotypes.

RAB-10 has been shown to function with the exocyst complex in vesicle trafficking and docking [23–25]. Therefore, we tested whether *rab-10* acts via the exocyst to regulate dendrite branch distribution in PVD. We examined PVD morphology in strains carrying mutations in each of several conserved exocyst genes, including *exoc-8* and *sec-5*. The *exoc-8(ok2523)* allele is a 1474 base pair deletion and a putative null [24]. The *sec-5(pk2358)* allele introduces an early stop codon at position 389, and has been described as a non-null, strong loss-of-function allele [42]. We found that mutations in either *exoc-8(ok2523)* or *sec-5(pk2358)* resulted in a posterior loss of dendritic branch complexity, nearly phenocopying *rab-10* mutants (Fig 5A and S2 Fig). The reduction in secondary branches in the +2 region was less dramatic in the *exoc-8(ok2523)* mutant than in the *rab-10* mutants (S1 Table), but significant reduction in branch complexity was observed in the +2, +1, and -1 regions (Fig 5A and 5F). *sec-5(pk2358)* displayed a less severe but qualitatively similar phenotype (S2 Fig). No significant 2° branch loss was observed in *sec-5* (S1 Table), but 4° branches were reduced in the +2, +1, and -1 regions (S1 Table). To test whether the exocyst complex functions cell-autonomously to pattern the PVD dendrite, we expressed *exoc*-8 using a PVD specific promoter in the *exoc-8(ok2523)* background. Expressing *exoc-8* in PVD rescued the dendritic morphology defect (Fig 5B and 5F), suggesting that *exoc-8* also functions cell-autonomously.

The striking anterior shift of dendritic complexity observed in *rab-*10 was not observed in *exoc-8(ok2523)* or *sec-5(pk2358*) mutants (Fig 5A and S2 Fig). While both *rab-*10 and the exocyst are required for branching in the normal +2,+1, and -1 regions, this indicates that *rab-*10’s role in anterior-posterior patterning may not require the exocyst components. We investigated the relationship between *rab-*10 and the exocyst with two genetic interaction experiments. Since *rab-10;exoc-*8 animals do not survive [24], we overexpressed the dominant-negative RAB-10(GDP) in *exoc-8* to assess the double mutant phenotype. Overexpression of RAB-10(GDP) in *exoc-8* further reduced branch complexity to *rab-10* levels and also caused an anterior shift in branch complexity, phenocopying *rab-10* (Fig 5E). The *exoc-8;*RAB-10(GDP) phenotype was indistinguishable from that of *rab-10* and not stronger than the *rab-10* phenotype (Fig 5G), suggesting that *rab-10* and the exocyst function in a common pathway. To further explore this genetic relationship, we overexpressed RAB-10(GTP) in the *exoc-8* background. RAB-10(GTP) was able to rescue the +2, +1, and -1 reduction in branch complexity caused by the *exoc-8* mutation (Fig 5C and 5F), suggesting that *rab-10* acts downstream of *exoc-8*. Together these data demonstrate that both *rab-10* and the exocyst act cell-autonomously to pattern the PVD dendrite. *rab-10* likely has *exoc-8* independent functions in anterior-posterior patterning, but these genetic interaction data indicate that *rab-10* and the exocyst function in a common pathway to promote proximal dendritic growth.

### DMA-1 localization is altered in *rab-10* mutants

The lack of stabilized dendritic branches, despite secondary filopodia outgrowth, in the *rab-10* mutants is similar to phenotypes found in the *dma-1* mutants [32–33]. We have previously demonstrated that DMA-1 functions cell autonomously in PVD as the branching receptor that responds to extrinsic SAX-7/MNR-1 guidance cues to promote 3° and 4° branch formation as well as stabilization of 2° branches [33]. We observed that in *dma-1* mutants, all 4° and most 3° branches are lost in all regions of PVD (S3 Fig and S1 Table). Since DMA-1 is a transmembrane protein and functions as a receptor on the plasma membrane, we hypothesized that *rab-10* and *exoc-8* might regulate the membrane localization of DMA-1.

**Fig 6:**
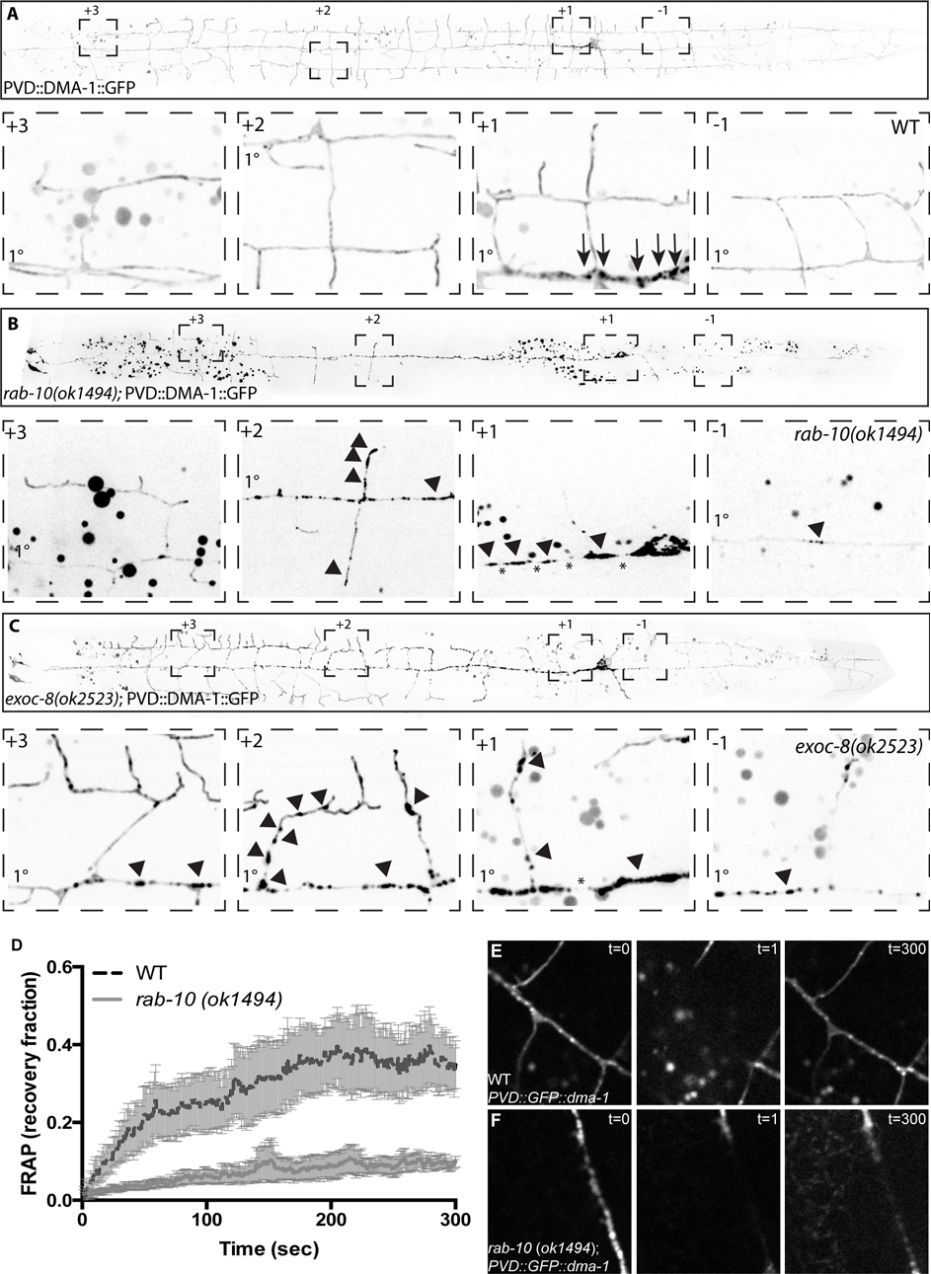
DMA-1 localization is disrupted in *rab-10* mutants. (A-C) Representative images of DMA-1::GFP in wild-type (A), *rab-10(ok1494*) (B), and *exoc-8(ok2523)* animals (C). Zoom-in images are shown from four regions in all genotypes. Arrows indicate DMA-1::GFP puncta, arrowheads indicate abnormal accumulations, and asterisks indicate regions from which the diffuse staining is lost. The primary dendrite is indicated on each zoom-in image. (D) Fluorescence recovery after photobleaching (FRAP) of DMA-1::GFP in wild-type and *rab-10(ok1494)* animals; error bars are SEM. (E,F) Three frames of fluorescent images during the FRAP experiment in wild-type (E) and *rab-10(ok1494)* animals (F).

To understand how RAB-10 and EXOC-8 regulate DMA-1 localization, we expressed a DMA-1::GFP fusion protein under the control of a PVD specific promoter in wild-type, *exoc-8* and *rab-10* animals. In wild-type animals, DMA-1::GFP displays a predominantly diffuse staining pattern throughout the entire PVD dendrite, which likely represents DMA-1 localization on the plasma membrane. DMA-1::GFP can also be observed as punctate structures that likely represent secretory and endocytic vesicles (Fig 6A). The majority of DMA-1 puncta co-localize with the late endosomal marker RAB-7, suggesting that they represent RAB-7-positive vesicles targeted for lysosomal degradation (S4 Fig). This co-localization is not disrupted in *rab-10*, but RAB-7 forms larger accumulations than the normal wild-type puncta in the primary dendrite of *rab-10* (S4 Fig). A small portion of the DMA-1 puncta overlap with RAB-10 puncta (S4 Fig). In *rab-10*, the diffuse component of DMA-1::GFP appears absent from the posterior dendrite (+2, +1 and -1 regions) and the DMA-1::GFP puncta form larger accumulations than in wild-type, similar to the change seen in RAB-7 localization (Fig 6B and S3 Fig and S4 Fig). In the anterior (+3) region of PVD, where the full dendritic arbor forms normally, DMA-1::GFP localization appeared largely normal in the *rab-10* mutant (Fig 6B). Since both *exoc-*8 and *rab-*10 are required for posterior dendritic growth, we also examined DMA-1::GFP localization in *exoc-*8. Similar defects in DMA-1::GFP localization were observed, with a decrease in diffusive staining in the +1 region and large accumulations of intracellular material in the primary and defective secondary branches. (Fig 6C).

To further examine this localization defect, we used fluorescence recovery after bleaching (FRAP) assays to examine the lateral mobility of DMA-1::GFP. We reasoned that the DMA-1 on the plasma membrane should be more diffusive compared with the DMA-1 trapped in intracellular membrane organelles. We focused on the primary dendrite in the +1 region because this region showed pronounced differences between wild-type and *rab-10* in both DMA-1::GFP localization and dendritic arbor formation. Indeed, wild-type animals showed a more complete and faster recovery of fluorescence than in the *rab-10* mutants (Fig 6D–6F), further supporting the notion that DMA-1 fails to be inserted into the plasma membrane in the posterior dendrite of the *rab-10* mutants.

In *dma-1* mutants, the PVD dendrite is almost completely devoid of 2°, 3°, and 4° branches [32–34] (S3 Fig), making the branching defect more severe than that of *rab-10*. This implies that DMA-1 function is not completely dependent on *rab-10*. Conversely, if *rab-10* controls PVD dendrite morphogenesis only by regulating DMA-1, we would predict that the *dma-1; rab-10* double mutant should phenocopy the *dma-1* mutant. In the *dma-1;rab-10* double mutant, 3° and 4° branches are lost in all regions of PVD, including the distal regions (S3 Fig and S1 Table), and in this regard it is indistinguishable from the *dma-1* single mutant. However, the double mutant has fewer 2° branches than either *rab-10* or *dma-1* alone (S3 Fig and S1 Table). This further reduction in 2° branches suggests that *rab-10* may regulate a larger cohort of proteins, which includes DMA-1, that are important for branching activity. To assess *rab-10*’s general role in distributing membrane proteins, we also examined the exogenous membrane protein mcd8.3 in both wild-type and *rab-10* animals and observed a similar phenomenon. More accumulations of large puncta, which likely represent intracellular protein, occurred in *rab-10* than in wild-type, and the diffuse proportion was reduced. This defect was most notable in the incomplete secondary branches (S3 Fig).

The normal subcellular localization of DMA-1 and excessive branches in the anterior dendrite of the *rab-10* mutant argue strongly that RAB-10 is not essential for the function of DMA-1. Instead, RAB-10 is required for correctly distributing “branching activity” along the anterior-posterior axis of the primary dendrite. This branching activity includes both DMA-1 and other factors required for dendrite branching. Even in the wild-type animals, the branches are not evenly distributed across the entire 1° dendrite: the anterior +3 region contains far fewer branches compared with the +2 or +1 region, suggesting a regulated distribution of branching activity (Fig 1B and 1H). It is therefore plausible that RAB-10 is critical to establish this distribution.

### Microtubule organization is uniform along the A-P axis of PVD

**Fig 7:**
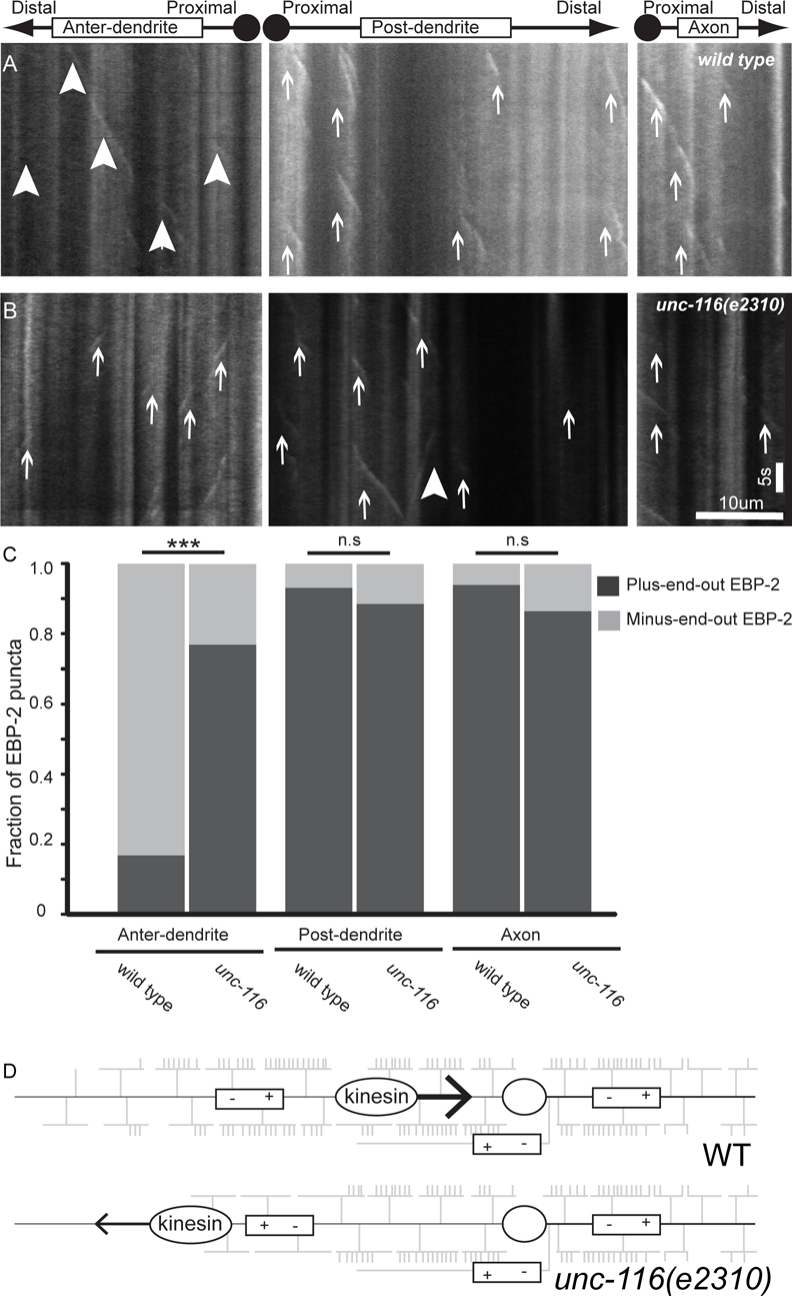
MT polarity is reversed in *unc-116/*kinesin1 mutants. (A,B) Representative EBP-2::GFP kymographs in different dendritic and axonal regions in wild-type (A) and *unc-116(e2310)* (B) animals. (C) Quantification of the direction of EBP-2::GFP comets. (D) Schematic of MT polarity and motor directionality in wild-type and *unc-116* PVD dendrite. Scale bars represent 10 μm and 5s. ***p<0.001. N>10 for all genotypes. Large arrowheads indicate retrograde movements, and small arrows indicate anterograde movements.

Since microtubule-based transport is essential for organelle and protein localization in large cells like PVD, we characterized the microtubule organization along the primary dendrite. Using the established EBP-2::GFP marker [43] to visualize the growing tips of microtubules in different regions of PVD, we found that microtubules in the anterior primary dendrite (+1, +2 and +3 regions) are predominantly oriented minus-end out with respect to the soma, with the minus end pointing to the distal anterior dendrite (Fig 7A and 7C). This is consistent with the literature that suggests most invertebrate dendrites adopt minus-end out microtubule organization [44–46]. Surprisingly, microtubules in the posterior dendrites (-1 region) are mostly plus-end out (Fig 7A and 7C), mimicking the organization of polarity in axons (Fig 7A and 7C). This microtubule organization dictates that a retrograde minus-end-directed motor, such as dynein, should move processively from the posterior-most dendrite to the anterior-most dendrite. Conversely, an anterograde plus-end-directed motor, such as kinesin-1, would move from the anterior-most dendrite to the posterior-most region.

**Fig 8:**
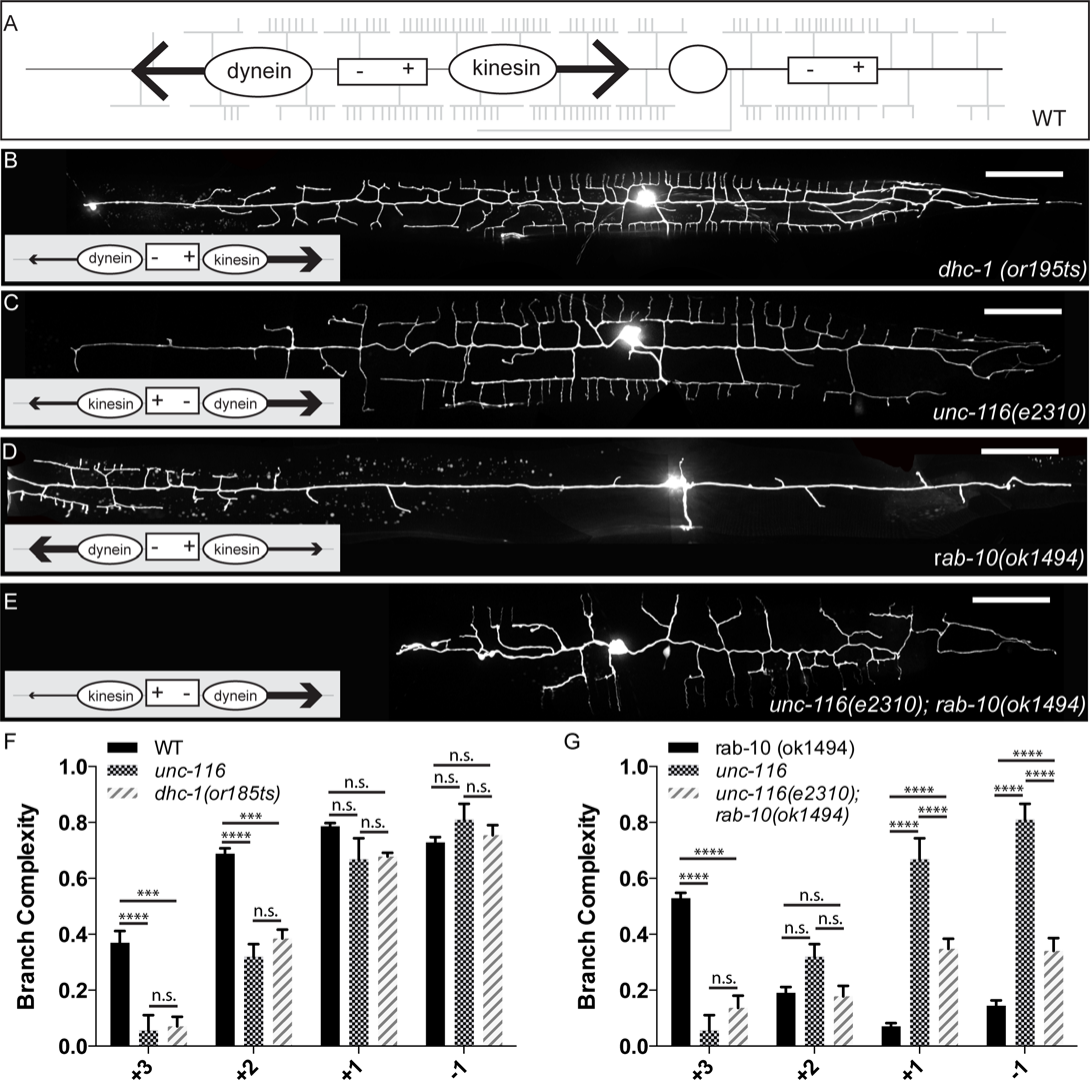
Molecular motors are required to establish anterior-posterior dendritic patterning. (A) Schematic of the directionality of motor movement based on MT polarity in a wild-type PVD dendrite. (B-E) Representative images of PVD::GFP in various genotypes, as indicated on each micrograph. Insets are schematics of MT polarity in the primary dendrite. (F) Quantification of subcellular distribution of branch complexity in wild-type, *unc-116(e2310)*, and *dhc-1(or195ts)* animals. (G) Quantification of subcellular distribution of branch complexity in *unc-116(e2310)*, *rab-10(ok1494)*, and *unc-116(e2310);rab-10(ok1494)* double mutant animals. Scale bars represent 20 μm. Error bars represent SEM. * p<0.05, **p<0.01, ***p<0.001, ****p<0.0001 by 2-way ANOVA with Tukey’s multiple comparisons test. N>5 for all genotypes.

We examined the PVD phenotype in *dhc-1* and *unc-116*/kinesin-1 mutants to directly test if major molecular motor systems are involved in PVD dendrite branching, possibly by transporting vesicles carrying DMA-1 and other dendrite outgrowth factors into the developing dendrite. Similar to previous a report, we found that the *dhc-1* mutant lacked dendritic branches specifically in the anterior region [36]. This is consistent with what we would expect based on microtubule organization in PVD, because dynein transports cargoes towards the anterior dendrite. Reduced dynein activity in the *dhc-1* mutant might result in the specific reduction of branching activity in the distal anterior dendrite. Strikingly, consistent with our predictions, the posterior dendritic branches showed no reduction in complexity in the *dhc-1* mutant (Fig 8B).

Next, we examined the *unc-116*/kinesin-1 mutant for PVD dendrite morphology defects. Similar to previous reports, we found that a partial-loss-of-function *unc-116* allele (*e2310*) caused a severe lack of anterior branches [36]. This phenotype is especially pronounced in the distal anterior region (Fig 8C). Superficially, this result is not consistent with our model; UNC-116/kinesin-1 should traffic cargoes toward the posterior part of the cell. However, our previous experiments in the neuron DA9 showed that UNC-116 is required for establishing microtubule polarity in the dendrite in addition to its known function in trafficking organelle cargoes. In the *unc-116* mutant, the DA9 dendrite adopts an axon-like “plus-end distal” organization, while the axonal microtubules are not affected [43]. To test if the *unc-116* mutant also alters microtubule polarity in the PVD dendrite, we visualized EBP-2::GFP comets in anterior and posterior primary dendrites. Similar to the result seen in the DA9 dendrite, the microtubules in the anterior PVD dendrites are reversed in orientation compared to the wild-type controls: the microtubules of the anterior dendrite switch from minus-end distal in wild-type to predominantly plus-end distal in the *unc-116* mutant (Fig 7B, 7C and 7D). Interestingly, posterior dendrites in the mutant maintain the plus-end distal orientation, similar to the wild-type control (Fig 7B, 7C and 7D). As a result of the polarity change in the partial-loss-of-function allele, kinesin/UNC-116 should traffic cargoes to the distal anterior and distal posterior dendrite, while dynein should traffic cargoes toward the cell body (Fig 7D). Therefore, in the *unc-116* mutants, both the reversal in microtubule polarity and the reduced activity of the kinesin/UNC-116 motor might contribute to the dendritic phenotype. It is conceivable that the reduced kinesin activity, normal dynein activity, and plus-end distal microtubule orientation leads to a specific loss of branching cargo from the anterior distal dendrite.

### RAB-10 regulates the dendritic trafficking of branching factors along the A-P axis

Together, these data demonstrate that microtubules in both anterior and posterior primary dendrite in wild-type animals are oriented such that the vast majority of minus-ends of microtubules point to the anterior end of the animal (Fig 7A). The dramatic anterior shift of dendritic arbor observed in *rab-10* suggested that RAB-10 could be responsible for patterning the dendritic arbor by directing protein trafficking along the A-P axis. It has previously been demonstrated that vertebrate Rab10 interacts with kinesin-1 to regulate axonal vesicle transport in the growing axon of hippocampal neurons [21]. Based on the anterior bias in branch distribution observed in the *rab-10* mutants, we hypothesized that RAB-10 promotes the transport of branching activity towards the plus ends of microtubules. To further test this hypothesis, we explored the genetic interaction between *rab-10* and *unc-116*. The reversed polarity of microtubules in the anterior dendrite of *unc-116* mutants provided a framework to test the potential cooperation between *rab-10* and *unc-116*. If the anterior shift of PVD dendritic branches seen in *rab-10* occurred as a result of a failure of plus-end directed trafficking, then we would expect the branching defect to be reversed by switching microtubule polarity: our hypothesis predicts that the double mutants should lack distal anterior dendrites but have branches in the anterior +1 and posterior -1 region. Indeed, we found that the double mutant showed reduced branch complexity in the +3 region compared with that of *rab-10* single mutants (Fig 8E). In striking contrast to the *rab-10* single mutants, which have their highest branch complexity in the anterior “+3” region, the double mutant has the highest branch complexity in the posterior “+1” and “-1” regions, where the microtubule minus-ends are directed (Fig 8E and 8G).

**Fig 9:**
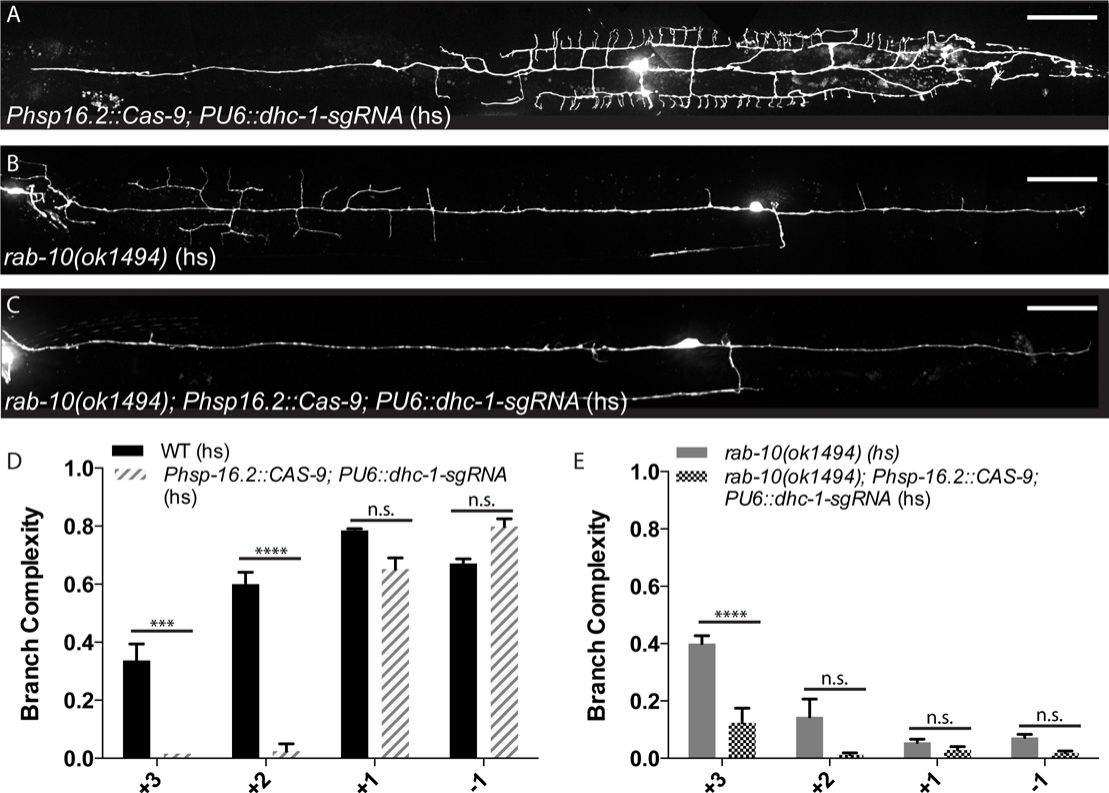
DHC-1 is required to distribute anterior branching activity. (A-C) Representative images of PVD::GFP in the conditional *dhc-1* CRISPR allele in the wild-type background (A), a heat-shocked *rab-10* control (B), and the *dhc-1* CRISPR allele in the *rab-10* background (C). (D) Quantification of subcellular distribution of branch complexity in the *dhc-1* CRISPR allele in the wild-type background. (E) Quantification of subcellular distribution of branch complexity in the *dhc-1* CRISPR allele in the *rab-10* background. Scale bars represent 20 μm. Error bars represent SEM. * p<0.05, **p<0.01, ***p<0.001, ****p<0.0001 by 2-way ANOVA with Tukey’s multiple comparisons test. N≥3 for all genotypes.

To further explore the relationship between *rab-10* and the molecular motors, we examined the effect of knocking down dynein in the *rab-10* background. In our model, the minus-end-directed dynein motor is predicted to be responsible for distributing branch activity to the distal anterior dendrite, and our hypothesis predicts that the loss of dynein would suppress the anterior-shifted branches of *rab-10*. We utilized the previously described somatic CRISPR-Cas9 system to knock down *dhc-1*. The conditional *dhc-1* knockout in the wild-type background resulted in a reduction of branch complexity in the distal anterior region, similar to the *or195ts* allele (Figs 8B, 9A and 9D). The conditional *dhc-1* knockout in the *rab-10* background resulted in a complete loss of branches from the distal anterior region (Fig 9C and 9E) in 57% of animals, consistent with our hypothesis that dynein is required for distributing distal branching activity. These genetic interaction experiments between RAB-10/UNC-116 and RAB-10/DHC-1 provide compelling support for RAB-10’s role in regulating motor-assisted transport of factors required for branching activity, and this suggests that RAB-10 is responsible for determining not just outgrowth, but also the distribution of branches.

We propose that RAB-10 plays dual functions in regulating dendritic branching. On one hand, it regulates dendritic trafficking of branching factors towards the plus-ends of microtubules along the A-P axis, likely through association with UNC-116/kinesin-1. The factors regulated by RAB-10 include the branching receptor DMA-1 and other unidentified membrane components. In addition, once the vesicles are delivered to the proper domain of the plasma membrane, RAB-10 is required to dock and fuse membrane vesicles through its interaction with the exocyst complex.

## Discussion

Many intracellular processes must work together during the construction of an elaborately branched, polarized neuron. The polarity of neurites must be appropriately established to allow directional transport, and factors required for dendrite growth and branching must be correctly sorted into transport vesicles. Molecular motors then transport these cargoes to appropriate subcellular loci, where vesicle docking and membrane exocytosis ensue. Disrupting any of these processes could have profound effects on dendritic morphogenesis.

We identified novel roles for the small GTPase RAB-10 and the exocyst complex in dendrite morphogenesis of the *C*. *elegans* PVD neuron using genetic approaches. Additionally, we found that the molecular motors kinesin and dynein are important for anterior-posterior patterning of the PVD dendritic arbor, and demonstrated genetic interactions between both RAB-10/kinesin-1 and RAB-10/dynein in patterning the anterior-posterior distribution of branches. We propose a two-part role for RAB-10 in dendrite morphogenesis. First, RAB-10 acts to pattern the dendritic arbor by promoting the trafficking of branching factors toward the plus-ends of microtubules. Second, RAB-10 promotes dendritic outgrowth by mediating docking and exocytosis of protein cargoes.

### Polarized trafficking underlies branch patterning in the PVD dendrite

Dramatic dendritic morphogenesis defects were observed in the posterior regions of PVD in the *rab-*10 mutant, which were also independently described in a recent report [38]. However, our detailed analysis of PVD morphology in *rab-10* mutants revealed an additional anterior-posterior patterning defect that has not been described previously. While branches are lost from the posterior dendrite, more exuberant branching is observed in the anterior distal dendrite compared with wild-type dendrites. This anterior shift of the dendritic arbor indicates that RAB-10 is not merely responsible for branch outgrowth but also for regulating the distribution of branching activity along the dendrite, a process we hypothesized to be mediated by molecular motors. Indeed, we found dendrite morphology defects in both dynein and kinesin-1 mutants. Based on the MT polarity in each genotype and their corresponding dendritic morphology phenotypes, we propose that RAB-10 potentiates UNC-116/kinesin-1 mediated transport, which moves cargoes from the anterior to posterior dendrite. This model is further supported by the observed phenotype in the *unc-116; rab-10* double mutant, which showed a suppression of the posterior branching defects seen in *rab-10*.

Our work indicates that PVD achieves its stereotyped dendritic branching pattern by balancing anterograde and retrograde transport of branching factors, which requires the correct establishment of microtubule polarity, the appropriate balance of motor activity, and the correct association of transport cargoes with these motors. We propose a model in which RAB-10 is required for this balance of motor activity. First, microtubule polarity in the anterior and posterior primary dendrites dictates that kinesins, such as UNC-116, traffic membrane organelles from the anterior to posterior dendrite. Dynein does the opposite. In wild-type animals, these two motors have a particular equilibrium of activity, which results in the “normal” distribution of branching factors. Second, the kinesin activity is slightly stronger than the dynein activity in PVD, which sets up a subtle posterior-anterior gradient with fewer branches in the distal anterior dendrite. Third, RAB-10 promotes kinesin-1 mediated trafficking. In the *rab-10* mutants, this model predicts that kinesin-1-mediated transport of branching factors is reduced, and dynein activity dominates, resulting in a shift of the distribution of branching factors towards the anterior dendrite. This shift is dependent on microtubule polarity, as demonstrated by the *unc-116; rab-10* double mutant, where microtubule polarity is reversed. In this mutant, dynein functions as a retrograde motor, which rescues dendritic branches in the posterior region.

We demonstrate a genetic interaction between RAB-10 and kinesin-1, and a physical interaction has been previously shown in hippocampal axons, where Rab10 mediates anterograde trafficking by associating with kinesin-1 and its adaptor protein JIP1 [21]. In PVD, RAB-10 may similarly regulate the balance of motor activity by directly activating UNC-116. Another possibility is that RAB-10’s role in balancing motor activity arises from a preferential association of RAB-10-positive plasmalemmal precursor vesicles with kinesin over dynein or that RAB-10 is involved in the appropriate sorting of kinesin and dynein cargos. Additionally, it has previously been shown that reduction of either dynein or kinesin-1 activity in Drosophila sensory neurons results in the loss of distal dendrites, suggesting that across many species, balancing motor activity is necessary to distribute branching components across developing dendrites [47]. Future research will focus on further characterization of the relationship between RAB-10 and the molecular motors to establish the nature of the genetic interactions between RAB-10/UNC-116 and RAB-10/dynein in the growing PVD dendrite.

### Docking and secretion of branching factors supports dendritic outgrowth

In addition to RAB-10’s role in patterning the PVD dendrite, we have also demonstrated that RAB-10 is required for branch outgrowth and the subcellular localization of transmembrane proteins such as DMA-1. This suggests a second role for RAB-10, in which RAB-10 and the exocyst coordinate docking and secretion of membrane and protein cargoes.

RAB-10 has previously been shown to work in conjunction with the exocyst complex in both endocytic trafficking and tracheal outgrowth [23–25]. In the PVD dendrite, we found that the loss of two exocyst complex proteins, EXOC-8 and SEC-5, results in reduced dendritic complexity in the posterior regions of PVD, nearly phenocopying *rab-10*. We also found that the constitutively active RAB-10(GTP) was able to rescue the dendritic phenotype of *exoc-8*, suggesting that *rab-10* and *exoc-8* act in a common pathway to support dendritic branch formation. Additionally, we demonstrated that a transmembrane protein responsible for dendrite growth and branching, DMA-1, was disrupted in *rab-10* mutants. These results suggest that RAB-10 and the exocyst complex play a role in the secretion of proteins that control dendritic outgrowth and branching. The requirement of RAB-10 and the exocyst for dendritic outgrowth and membrane protein localization were also described in a recent report [38], which adds independent support to our discovery that RAB-10 and the exocyst are required for dendritic branch morphogenesis. We therefore propose that RAB-10 and the exocyst act together to control dendritic outgrowth by supporting docking and exocytosis of protein and membrane cargoes at appropriate subcellular dendritic domains.

Taken together, our data provide support for new roles for the small GTPase RAB-10 in dendritic outgrowth and patterning. RAB-10 coordinates the distribution of branching factors along the A-P axis of the growing PVD dendrite, and in conjunction with the exocyst complex, RAB-10 allows for the docking and secretion of branching factors such as DMA-1. Our work provides novel insight into the regulation of branch distribution by RAB-10 and demonstrates that the regulation of dendritic trafficking by Rab GTPases is of critical importance in the establishment of complex dendritic arbors.

## Methods

### Strain maintenance and genetics

Worms were raised on OP50 *Escherichia coli*-seeded nematode growth medium plates at 20°C [48]. The wild-type reference strain was N2 Bristol. DV2689 *sec-5*(*pk2358*)/mln[*dpy-10*(*e128*)mls14], RB1928 *exoc-8* (*ok2523)*, and VS1026 *rab-10* (*ok1494*) were obtained from the *Caenorhabditis* Genetics Center.

### Isolation and mapping of the *wy787* mutant allele

The wy787 allele was isolated from an F2 semiclonal screen of 3,000 haploid genomes in the PVD::myrGFP strain. Worms were mutagenized with 50mM ethyl methanesulfonate. The *rab-10* locus was identified with SNP mapping, fosmid rescue, and sequencing.

### Transgenes

Two PVD-specific promoters, *ser2prom3* and *pdes2short* [30], were used interchangeably for PVD specific expression; *phlh-1* was used for muscle-specific expression. The integrated wyIs592 (*ser2prom3*::*myrGFP*) and the extrachromosomal array *ser2prom3*::*myrmCherry* were used to visualize PVD. *wyEx4286* (*ser2prom3*::*dma-1*::*GFP*) was used to visualize DMA-1 localization. *wyEx4985* (*pdes2*::*ebp-2*::*GFP*) was used to visualize plus-end microtubules. Transgenes generated for this work include: *wyEx7698* (*ser2prom3*::*rab-10*), *wyEx7701* (*pdes2short*::*rab-10*::*GFP*), *wyEx7700* (*pdes2short*::*rab-10(GTP)*::*GFP*), *wyEx7772* (*pdes2short*::*rab-10(GDP)*::*GFP*), wyEx7763 (*ser2prom3*::*exoc-8*), *wyEx7699* (*hlh-1*::*rab-10*), *rab-10*::*rab-10*, *wyEx7069 (PVD*::*mcd8*.*3)*, *ser2prom3*::*rab-10*::*mCherry; wyEx7137 (ser2prom3*::*rab-7*::*mCherry)*, *wyEx50081 (Phsp16*.*2*::*Cas-9;PU6*::*dhc-1-sgRNA)*, and *Phsp16*.*2*::*Cas-9;PU6*::*denn-4-sgRNA*. *Podr-1*::*RFP* and *Punc-122*::*RFP* were used as coinjection markers.

### Fluorescence microscopy

Images were captured in live animals using a Plan-Apochromat 63x/1.4 objective on a Zeiss LSM710 confocal microscope or using a Plan-Apochromat 63x/1.4 objective on a Zeiss Axio Observer Z1 microscope equipped with a Yokagawa spinning disk head. For still images, worms were immobilized using 10 mM Levamisole (Sigma-Aldrich) in M9 on 2% agarose pads. For FRAP imaging of DMA-1::GFP and time-lapse imaging of EBP-2::GFP, worms were soaked in 0.1% tricane/0.01% levamisole in M9 for 20 minutes and were then mounted on 5% agarose pads with 0.05μg of polysterene microspheres (Polysceinces). Video for EBP-2::GFP comet analysis was acquired at 8 frames per second.

### Quantification of dendritic arbor

Images were straightened with the ImageJ software (US National Institutes of Health) using the primary dendrite as a reference. In L4 animals, the PVD dendrite was divided into four segments (three equal-length anterior segments, and one posterior segment) and the number of secondary and quaternary branches per segment was counted. Quaternary branches were assigned to a segment based upon the location of the secondary branch forming the base of the menorah from which the quaternary branch originated. Any protrusion from the primary dendrite was classified as a secondary branch, and any correctly-oriented protrusion from the tertiary dendrite was classified as a quaternary branch. A “branch complexity” index, defined in Fig 1, was computed for each segment. This term weights each branch order and is normalized to an “ideal” segment. In L2 and L3 animals, only the total number of secondary branches was tabulated.

### Somatic CRISPR-Cas9 experiments

CRISPR-Cas9 constructs were designed according to previously established methods [40]. *Phsp16*.*2*, a heat-shock promoter expressed in many tissues, was used to drive Cas9 expression. Worms were synchronized via bleaching and L1 plates were heat shocked at 33°C for two hours. The efficiency of the CRISPR-Cas9 editing was determined by observing >30 animals of each genotype. For the *denn-*4 experiment, which had ~20% efficiency, animals with the CRISPR-Cas9 array and an observed PVD phenotype were selected for imaging and quantification, in comparison to wild-type controls without the array from the same plate. For the *dhc-1* experiment, all animals with the CRISPR-Cas9 array were included for imaging and quantification, without pre-screening for neuronal phenotype, because the *dhc-1* sgRNA had a much higher efficiency (~60%). Animals from multiple replicates of each CRISPR experiment were pooled for the quantification.

### FRAP analysis

FRAP experiments were performed using the Zeiss Axio Observer Z1 microscope equipped with a Yokagawa spinning disk head. A small region of primary dendrite just anterior to the cell body was bleached for 1000ms. Recovery images were taken every second for five minutes.

### Statistical analysis

Branch complexity numbers per segment were compared across all genotypes using the 2-way ANOVA with post-hoc Tukey’s multiple comparisons test. Similarly, branch complexity numbers were compared across all genotypes within each somatic CRISPR experiment using the 2-way ANOVA with post-hoc Tukey’s multiple comparisons test. FRAP data were compared using 2-way ANOVA. Developmental differences in secondary branches between wild-type and *wy78*7 were assessed using multiple t-tests.

## Supporting Information

S1 Fig Active RAB-10 is cell-autonomously required for dendritic branching. (A) Schematic of conservation of the RAB-10 GTPase site, with homology across species and conservation between various Rabs. (B-C) Representative images of PVD::GFP in *rab-10(ok1494)* with expression of *rab-10* in muscle (B) or *rab-10* genomic DNA under its own promoter (C). (D-E) Representatives images of PVD::GFP in wild-type expressing an inactive, dominant negative GDP-bound RAB-10 in PVD, demonstrating the range of branch loss caused by DN RAB-10. Scale bars represent 20 μm.
(TIF)

S2 Fig Loss of sec-5, an exocyst complex component, disrupts dendritic morphology. (A) Representative image of PVD::GFP in *sec-5 (pk2358)*. (B) Quantification of subcellular distribution of branch complexity in the *sec-5(pk2358)* mutant animals. Scale bars represent 20 μm. Error bars represent SEM. * p<0.05, **p<0.01, ***p<0.001, ****p<0.0001 by 2-way ANOVA with post-hoc Tukey’s multiple comparisons test. N≥3 for all genotypes.
(TIF)

S3 Fig The distribution of membrane proteins is disrupted in *rab-10* mutants. (A-B) Representative images of PVD::GFP in various genotypes, as indicated on micrographs. (C) Quantification of subcellular distribution of branch complexity in the *rab-10(ok1494)*, *dma-1(wy686)*, and *dma-1(wy6860);rab-10(ok1494)* double mutant animals. (D) Quantification of DMA-1::GFP puncta size in *rab-10(ok1494)* and wild-type. (E-F) Representative image of mcd8.3::GFP in wild-type (E) and *rab-10(ok1494)* (F). Zoom-in images indicate mcd8.3::GFP localization in the +1 and +2 regions. Arrows indicate mcd8.3::GFP puncta, arrowheads indicate abnormal accumulations, and asterisks indicate regions from which diffuse staining is lost. The primary dendrite is indicated on each zoom-in image. Scale bars represent 20 μm. Error bars represent SEM. * p<0.05, **p<0.01, ***p<0.001, ****p<0.0001 by 2-way ANOVA with Tukey’s multiple comparisons test. N≥3 for all genotypes. Arrowheads indicate diffuse staining; arrows indicate puncta.
(TIF)

S4 Fig DMA-1 co-localizes with the endosomal marker RAB-7. (A-B) Representative images of DMA-1::GFP and mCherry::RAB-7 in the anterior primary dendrite of wild-type (A) and *rab-10(ok1494)* (B) animals. (C) Representative images of DMA-1::GFP and mCherry::RAB-10 in the anterior primary dendrite of wild-type animals.
(TIF)

S1 Table PVD branch numbers by genotype. The average number of branches per subcellular compartment and the total number of branches are reported for each genotype. Wild-type is listed first, with other genotypes listed in order of increasing total quaternary branches.
(XLSX)
