# Supplementary figures and images for: RAB-10 Regulates Dendritic Branching by Balancing Dendritic Transport
Source: PLoS Genet. 2015 Dec 3;11(12):e1005695. doi: 10.1371/journal.pgen.1005695 (PMC4669152; doi:10.1371/journal.pgen.1005695)

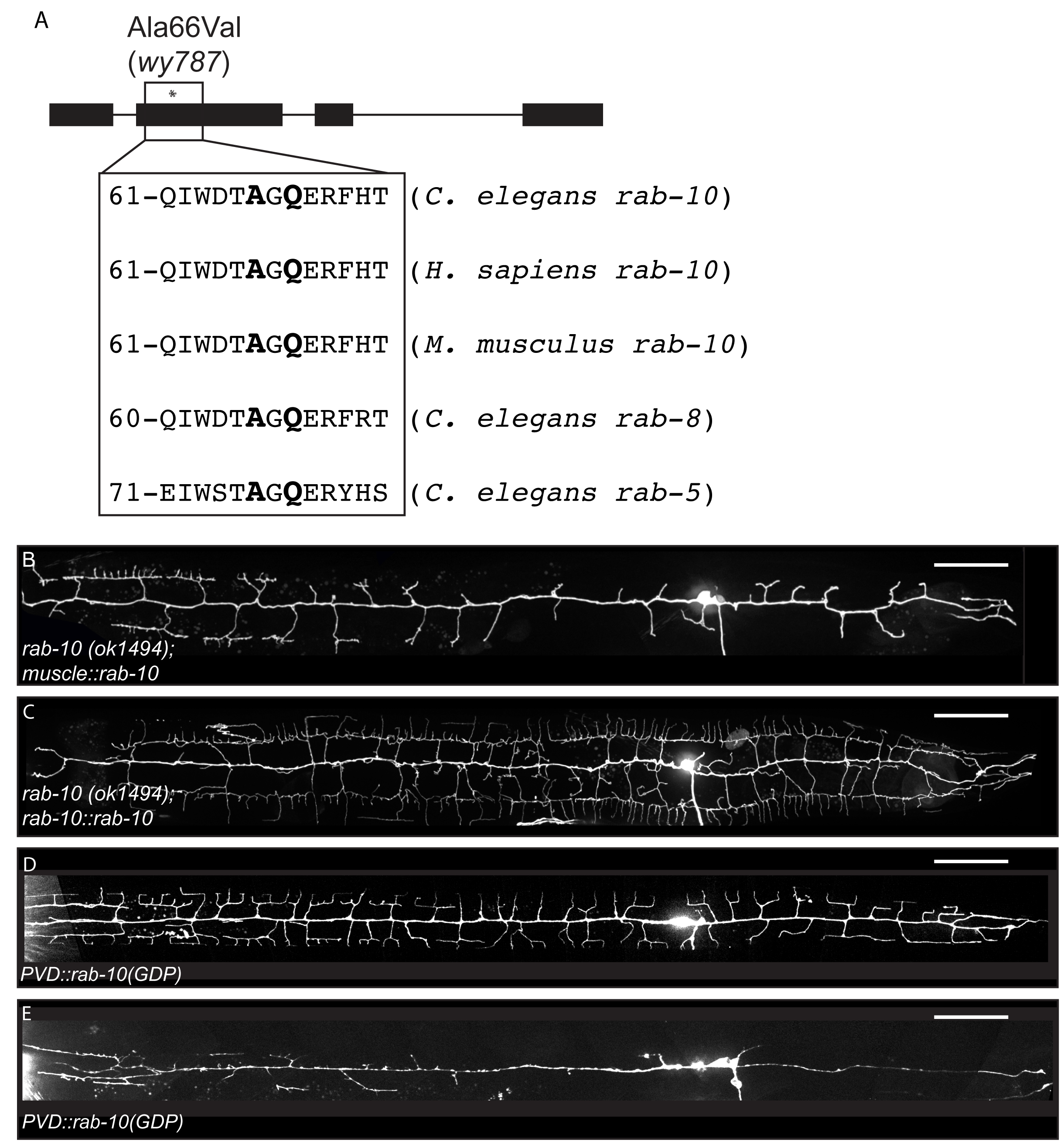

Supplement: S1 Fig — (A) Schematic of conservation of the RAB-10 GTPase site, with homology across species and conservation between various Rabs. (B-C) Representative images of PVD::GFP in rab-10(ok1494) with expression of rab-10 in muscle (B) or rab-10 genomic DNA under its own promoter (C). (D-E) Representatives images of PVD::GFP in wild-type expressing an inactive, dominant negative GDP-bound RAB-10 in PVD, demonstrating the range of branch loss caused by DN RAB-10. Scale bars represent 20 μm. (TIF) [file pgen.1005695.s001.tif]

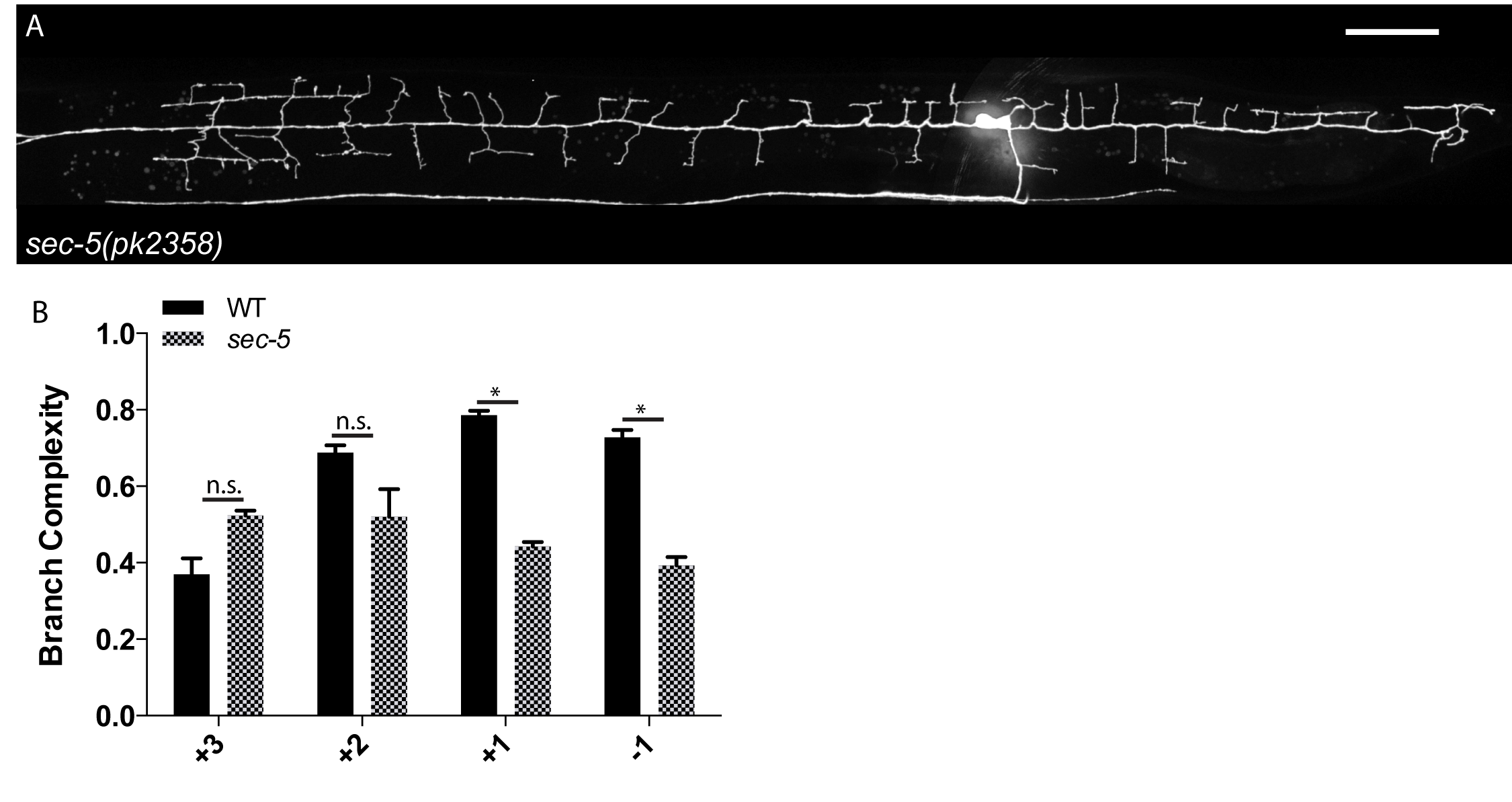

Supplement: S2 Fig — (A) Representative image of PVD::GFP in sec-5 (pk2358). (B) Quantification of subcellular distribution of branch complexity in the sec-5(pk2358) mutant animals. Scale bars represent 20 μm. Error bars represent SEM. * p<0.05, **p<0.01, ***p<0.001, ****p<0.0001 by 2-way ANOVA with post-hoc Tukey’s multiple comparisons test. N≥3 for all genotypes. (TIF) [file pgen.1005695.s002.tif]

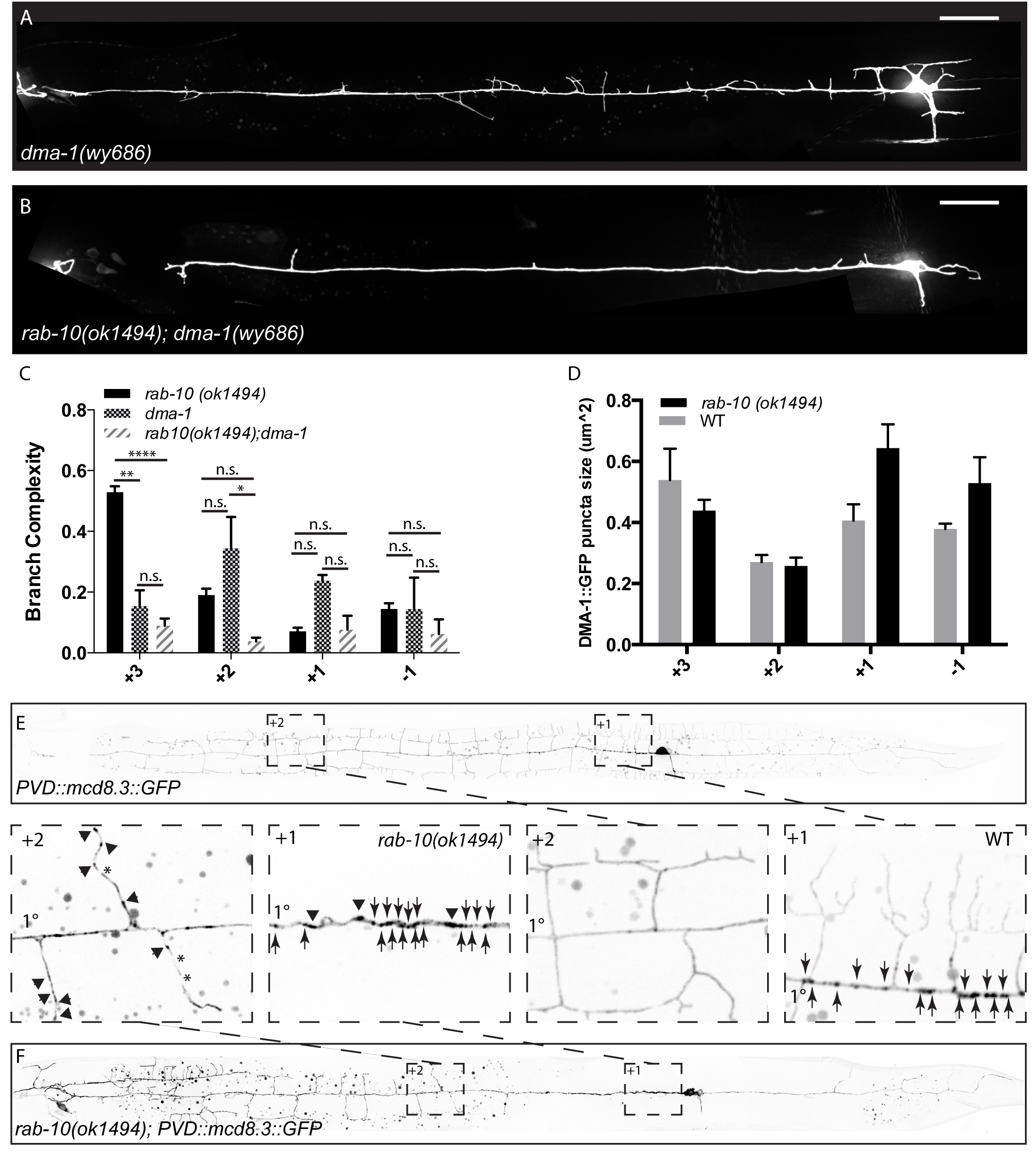

Supplement: S3 Fig — (A-B) Representative images of PVD::GFP in various genotypes, as indicated on micrographs. (C) Quantification of subcellular distribution of branch complexity in the rab-10(ok1494), dma-1(wy686), and dma-1(wy6860);rab-10(ok1494) double mutant animals. (D) Quantification of DMA-1::GFP puncta size in rab-10(ok1494) and wild-type. (E-F) Representative image of mcd8.3::GFP in wild-type (E) and rab-10(ok1494) (F). Zoom-in images indicate mcd8.3::GFP localization in the +1 and +2 regions. Arrows indicate mcd8.3::GFP puncta, arrowheads indicate abnormal accumulations, and asterisks indicate regions from which diffuse staining is lost. The primary dendrite is indicated on each zoom-in image. Scale bars represent 20 μm. Error bars represent SEM. * p<0.05, **p<0.01, ***p<0.001, ****p<0.0001 by 2-way ANOVA with Tukey’s multiple comparisons test. N≥3 for all genotypes. Arrowheads indicate diffuse staining; arrows indicate puncta. (TIF) [file pgen.1005695.s003.tif]

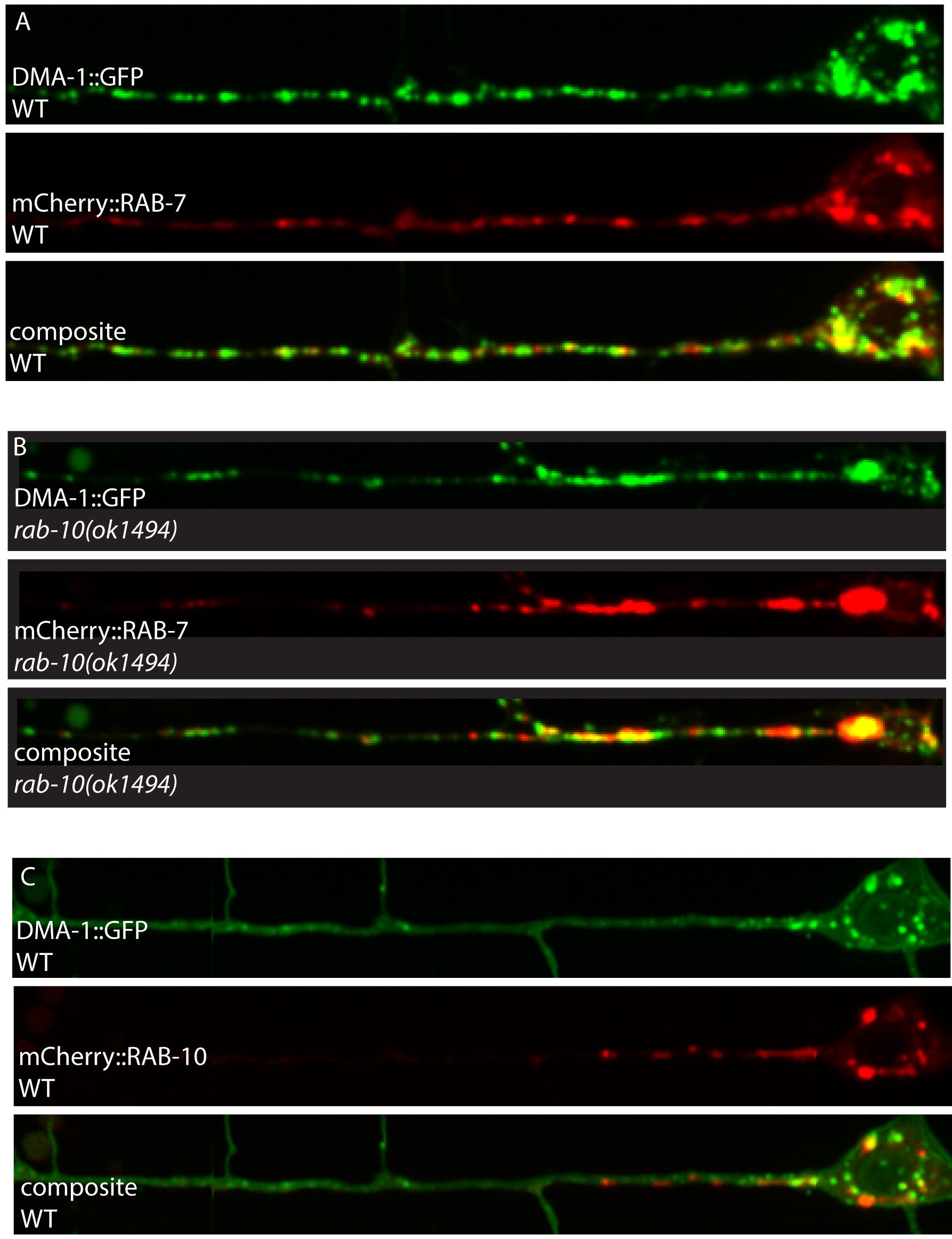

Supplement: S4 Fig — (A-B) Representative images of DMA-1::GFP and mCherry::RAB-7 in the anterior primary dendrite of wild-type (A) and rab-10(ok1494) (B) animals. (C) Representative images of DMA-1::GFP and mCherry::RAB-10 in the anterior primary dendrite of wild-type animals. (TIF) [file pgen.1005695.s004.tif]
